# Transient immunostimulation with LPS promotes tissue repair in aged skin

**DOI:** 10.1186/s12979-026-00570-y

**Published:** 2026-05-07

**Authors:** Philipp Haas, Yongfang Wang, Albert Kallon Koroma, Jinnan Cheng, Mahyar Aghapour, Adelheid Hainzl, Linda Krug, Susanne Schatz, Meinhard Wlaschek, Pallab Maity, Karin Scharffetter-Kochanek, Karmveer Singh

**Affiliations:** Department of Dermatology and Allergic Diseases, Ulm University, N27, Albert-Einstein-Allee 23, Ulm, 89081 Germany

## Abstract

Tissue repair is often hampered during aging. Worldwide, chronic wounds in elderly present a major challenge to the medical and socioeconomic infrastructure of societies. A comprehensive understanding of how the aging innate immune system impacts wound homeostasis is lacking. Here we employed the approach of immune modulation to restore disrupted wound repair in aged mice skin. We found that a short pulse of bacterial lipopolysaccharide (LPS) before wounding markedly accelerate tissue repair in aged mice, which – if non-primed – exhibit a defective epidermal wound closure. LPS priming induces rapid sealing of wounds, immune cell activity, keratinocyte responsiveness and their differentiation towards a newly reconstituted wound epithelium. Structural elements such as NETs composed of DNA and membrane protrusions derived from LPS-activated neutrophils and macrophages, respectively, reinforce physical skin barrier in aged wounds. The physical barrier established by LPS-primed innate immune cells subsequently facilitates epithelial tongue migration and adhesion of ECM-producing mesenchymal cells. Collectively, this not only prevents the invasion of pathogens into the restoring skin tissue after injury, but also averts the persistence of low-grade inflammation associated with aged wounds. These findings underscore the benefit of immune cell priming in promoting cellular interactions between innate immune cells and epithelial cells that consequently restores physical skin barrier and promote tissue repair.

**Supplementary Information:**

The online version contains supplementary material available at 10.1186/s12979-026-00570-y.

## Introduction

Aging is characterized by the functional decline and loss of structural integrity of all organs across species [1, 2]. In mammals, aging is accompanied by reduced tissue regeneration and persistent inflammation [3, 4]. Among other factors, age-associated mild, though lasting inflammation profoundly interferes with different stages of wound healing [3, 5].

Information on what accounts for the profound age-related decline in tissue regeneration and the persistence in the non-healing state of inflammation is not sufficient. Currently, chronic wounds present a substantial economic burden to the healthcare system and causes high morbidity and mortality in the large proportion of elderly population [6, 7].

Wound healing in mice closely reflects tissue repair in humans, which occurs in overlapping yet highly synchronized phases that includes blood clotting, inflammation, re-epithelialization and a long-lasting tissue remodeling phase [8, 9]. Organisms are protected from external threats by dedicated physical barriers that confer mechanical protection and prevents the entry of pathogens. If microbes enter the organism, another layer of innate immune defense becomes active to kill the invading microorganisms. Successful tissue repair requires a well-orchestrated sequence of proliferation, migration and differentiation of regenerative stem cells pools and their coordination with other tissue resident cells and immune cells recruited to the site of tissue injury [9, 10]. Restoration of tissue homeostasis after injury is largely dictated through bidirectional communication between epithelial cells and immune cells [11]. However, this highly programmed tissue repair may be negatively affected at various steps during aging, and this eventually results in the development of the non-healing state of chronic wounds. Tissue repair during aging comprise alterations in extracellular matrix deposition, mechanical forces, chemokine and cytokine release all potentially contributing to healing impairment [1, 12, 13].

Large skin lesions result in a severe breach of the epidermal barrier with the disruption of different keratinocyte layers and discontinuation of the basement membrane beneath. This disruption of the epidermal barrier eventually leads to the loss of protection against environmental threats to internal organs [12]. Damaged skin barrier - if not repaired adequately - enforces infection, local tissue damage, continuous recruitment of immune cells and chronic wound formation [14]. Currently, there is only limited information on the impact of aging on the regulation of barrier-linked inflammation, unrestrained recruitment of innate immune cells and the aggravation of tissue damage.

In recent years, the emerging concept of immune memory that were originally thought to limited to adaptive immune system, was established also for innate immune cells such as macrophages, monocytes, natural killer cells and for non-immune cells such as hematopoietic stem cells and epithelial cells as well [15–18]. So far it is unclear whether priming with an inflammatory stimulus of immune cells leads to a response in the aged wound microenvironment.

In the present study, we investigated whether transient priming with the bacterial wall molecule LPS induces an acute response in immune cells and impacts tissue repair in old mice skin.

## Materials and methods

### Mice experiments

For wound healing studies young (2 months) and old (21 months) wild type (C57BL/6J) mice were used. Mice were directly purchased from Jackson laboratories and housed under pathogen free conditions at the animal care facility of Ulm University in compliance with the German Law for Welfare of Laboratory Animals. All experiments were approved by the RP Tübingen Baden-Württemberg (approval number TVA 1547). In wound healing experiments, mice were intraperitoneally (i.p.) injected either with vehicle (PBS) or 500ng/ml LPS 24 h prior to inflicting four full excisional thickness wound on their back skin using 6 mm punch biopsies. Wound closure kinetics were monitored as previously described [19]. For histological analysis wound tissues were harvested at different time points and fixed in 4% PFA.

### Histology and immunostaining

Histology and immunostainings were performed on 5 μm thick wound sections or sections from non-wounded skin biopsies and lung tissues by standard procedures as previously described [20, 21]. We employed primary antibodies against F4/80, Ly6G, CD206 (Biolegend), K10, K14, CD3 (e-Bioscience), Ki-67 (Thermo), IL-6 and α-SMA (R&D), Collagen 3 (Novus), Arginase-1, pSTAT3, Neutrophil elastase, IL-1β and Citrullinated histone H3 (Cell Signaling). Respective isotypes were used as negative controls, and AF488, AF555, and AF647conjugated (Life Technologies) secondary antibodies were employed. Nuclei were counterstained with DAPI. Following staining, tissues were mounted with fluorescence mounting medium (DAKO). Images were captured by Zeiss Axiophot microscope equipped with an AxioCam digital color camera, Zen 3.2 (ZEN lite) and AxioVision software v4.8 (Carl Zeiss).

### Flow cytometry

Flow cytometry assay from skin wounds was performed as described earlier [22]. Briefly, single cell suspensions from finely minced fresh wounds were perepared by incubating them with enzymatic digestion cocktail (0.350 mg ml − 1 liberase TL, 0.1 mg ml − 1 DNase in serum-free RPMI) for 30 min at 37 °C on a rocking platform. At the end, 0.5 ml of 0.25% Trypsin-1 mM EDTA was added into the digestion reaction and incubated further for 10 min and reaction was then stopped by adding 10% fetal calf serum. Cell suspesions were then filtered, washed and centrifuged to collect the cells. Live cells were then separated by emplyoing dead cell removal microbeads kit (Miltenyi biotec). Single cell suspesions were then stained with the fluorophore-conjugated antibody cocktails described in Table 1.

**Table 1:** List of antibodies used for FACS analysis. Follwoing staining sample were run on BD LSRII cytometer (BD Biosciences) and analyzed by the BD FACS Diva analyser suite (BD Biosciences). Visualization and further analysis of flow cytometry data were performed using FlowJo (Version 9) and FlowLogic (Version 1.0)

Antibody	Company	Catalogue No
PE Anti-Mouse CD3	BD Biosciences	553063
PE Hamster IgG2, κ Isotype Control	BD Biosciences	550085
Alexa Fluor^®^ 647 anti-mouse neutrophil elastase	Cell Signaling	79565
Alexa Fluor^®^ 647 Rabbit IgG Isotype Control	Cell Signaling	3452
Alexa Fluor^®^ 488 anti-mouse myeloperoxidase	Cell Signaling	52972
Alexa Fluor^®^ 488 Rabbit IgG Isotype Control	Cell Signaling	4340
Alexa Fluor^®^ 488 anti-mouse CD36	Biolegend	102607
Alexa Fluor^®^ 488 Armenian Hamster IgG Isotype	Biolegend	400923
PE/Cyanine7 anti-mouse CD206 (MMR)	Biolegend	141719
PE/Cyanine7 Rat IgG2a, κ Isotype	Biolegend	400521
APC anti-mouse/human CD11b	Biolegend	101212
APC Rat IgG2b, κ Isotype	Biolegend	400611
PE/Cyanine5 anti-mouse CD86	Biolegend	105015
PE/Cyanine5 Rat IgG2a, κ Isotype	Biolegend	400509
PE anti-mouse Arginase 1	Biolegend	165803
PE Rat IgG2b, κ Isotype	Biolegend	400635
Brilliant Violet 421™ anti-mouse CD45	Biolegend	103133
Brilliant Violet 421™ Rat IgG2b, κ Isotype	Biolegend	400639
PE/Cyanine7 anti-mouse F4/80	Biolegend	123113
PE/Cyanine7 Rat IgG2a, κ Isotype	Biolegend	400521

### Statistical calculations

Error bars represent SEM. The significance of differences between two groups was analyzed by Student’s t test or one-way ANOVA, followed by Bonferroni correction for comparing the difference between more than two groups and presented as absolute p values.

## Results

### Delayed re-epithelialization and prolonged inflammation in injured aged skin

**Fig. 1:**
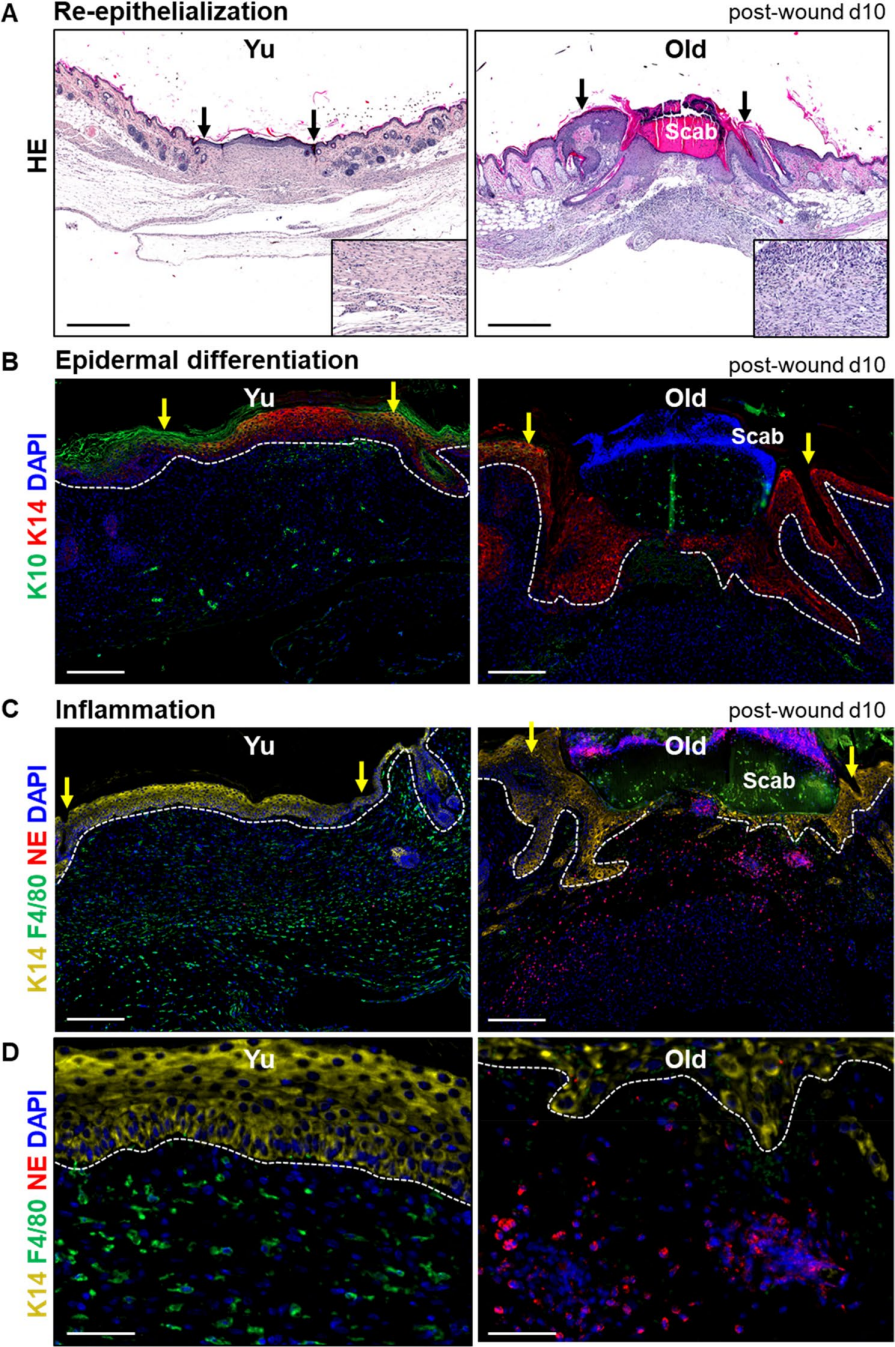
Aging impairs healing process at various phases. **A** Representative H&E photomicrographs depicting wound architecture in young and old mouse skin 10 days post injury. Arrows indicating wound edges. Scale bars, 500 μm. **B** Phase of re-epithelization and epidermal differentiation. Immunostaining of K14 (red), a keratin marker for basal epidermal keratinocytes, and K10 (green), a keratin marker for keratinocyte differentiation indicative for barrier formation in full thickness skin wounds of young and old mice. Nuclei stained with DAPI (blue). The stippled line indicates the epidermal-dermal junction and arrows indicating wound edges. Scale bar: 200 μm. **C**-**D** Inflammatory phase of wound healing. Immunostaining of neutrophil elastase a marker for recruited neutrophils (red) and F4/80 (green), a marker for macrophages in skin wounds of young and old mice. Nuclei stained with DAPI (blue). The stippled line indicates the epidermal-dermal junction and arrows indicating wound edges. Scale bar: 200 μm in C and 50 μm in D

Aging is linked to a higher susceptibility for infections and poor healing responses [15]. To explore impairment of wound repair during aging, four 6 mm full excisional thickness punch biopsies including whole epidermis and dermis were created on the back of young (2 months) and old (21 months) C57BL/6J mice. Wound sections were analyzed at 10 days after injury. Young mice consistently revealed a faster re-epithelialization when compared to wounds of aged mice (Fig. 1A). Wounds of aged mice depict impaired re-epithelialization and epidermal hyperplasia at the edges and a severe infiltration of the restoration site with inflammatory cells with lobulated nuclei as occurring in neutrophils (Fig. 1A). It is widely accepted that upon differentiation keratinocytes form a robustly sealed epidermal barrier that insulate skin from external insults [23, 24]. To further understand the age-dependent deficiency in re-epithelialization and terminal differentiation of keratinocyte in the newly forming wound epithelium, wound sections were stained with keratinocyte marker K14, a keratin indicative for proliferating and migrating keratinocytes and the keratinocyte keratin differentiation marker K10. Wounds of aged mice revealed defects in re-epithelialization and depicted epidermal thickening as opposed to wounds from young mice (Fig. 1B). In addition, terminal differentiation of keratinocytes (keratin K10) in the newly established wound epidermis of old mice was completely absent and only the immature keratin 14 was expressed at the wound edges (Fig. 1B). By contrast, there was a strong keratin 10 in the regenerating epithelium at the periphery and even at the center of wounds in young mice (Fig. 1B). These data highlight that during aging re-epithelialization and differentiation process is delayed in wounds.

Next, we analyzed the immune response, which is dysregulated during aging and results in a perpetuated inflammation eventually leading to inflammatory disorders [3]. To investigate whether inflammation persists at later stages of wound healing in old mice, we analyzed neutrophils and macrophages, two key cells of the innate immune system. Physiologically, neutrophils and pro-inflammatory macrophages are heavily recruited at the beginning of tissue repair to counteract infection and clear tissue debris [10]. Thereafter neutrophils are engulfed by macrophages and thereby initiate a switch toward an inflammation resolving regenerative macrophage subpopulation which enforces the phase of matrix deposition and remodeling [8, 25, 26]. At later stages of physiologically occurring wound healing neutrophils completely disappear [25, 27]. Interestingly, immunostaining of the neutrophil specific marker neutrophil elastase (NE) depicts that neutrophils persist at high numbers in wounds at 10 days after injury at the wound site of aged mice injury (Fig. 1C-D and Supplementary Fig. 1A). By contrast, the number of neutrophils already have significantly declined in wounds of young mice 10 days after injury (Fig. 1C-D and Supplementary Fig. 1A). Wound sections of young mice show significantly higher number of inflammation resolving F4/80 expressing macrophages compared to aged wounds 10 days post injury (Fig. 1C-D and Supplementary Fig. 1A). The F4/80 is widely used as classical monocyte/macrophage marker, recently single cell sequencing analysis suggest that these cells present in lower dermis and express the C1q^Hi^ M2 like marker [5], shown to participate in tissue remodeling and resolving inflammation [22, 28, 29]. In d5 wounds, we observed that F4/80 macrophages migrate from lower fascia and subcutaneous fat region and spread over to entire wound with time especially in young wounds (Supplementary Fig. 1B-C). These data, in fact, confirm their role rather in later tissue resolving phases and not in early pro-inflammatory cleaning phases of wound healing, while the macrophages subtype expressing arginase-1 dominated early stages of wound repair in old mice skin (Supplementary Fig. 1B-C). These arginase-1 positive macrophages were localized more closely to the epithelial barrier in wounds of young mice as opposed to wounds of old mice, however, similar numbers were found in both young and old d5 wounds (Supplementary Fig. 1B-C).

Taken together, these findings suggest a disturbed re-epithelialization and delayed immune response likely enforced neutrophil-dominated inflammation in wounds of aged mice.

### LPS priming accelerates tissue repair in aged skin

**Fig. 2:**
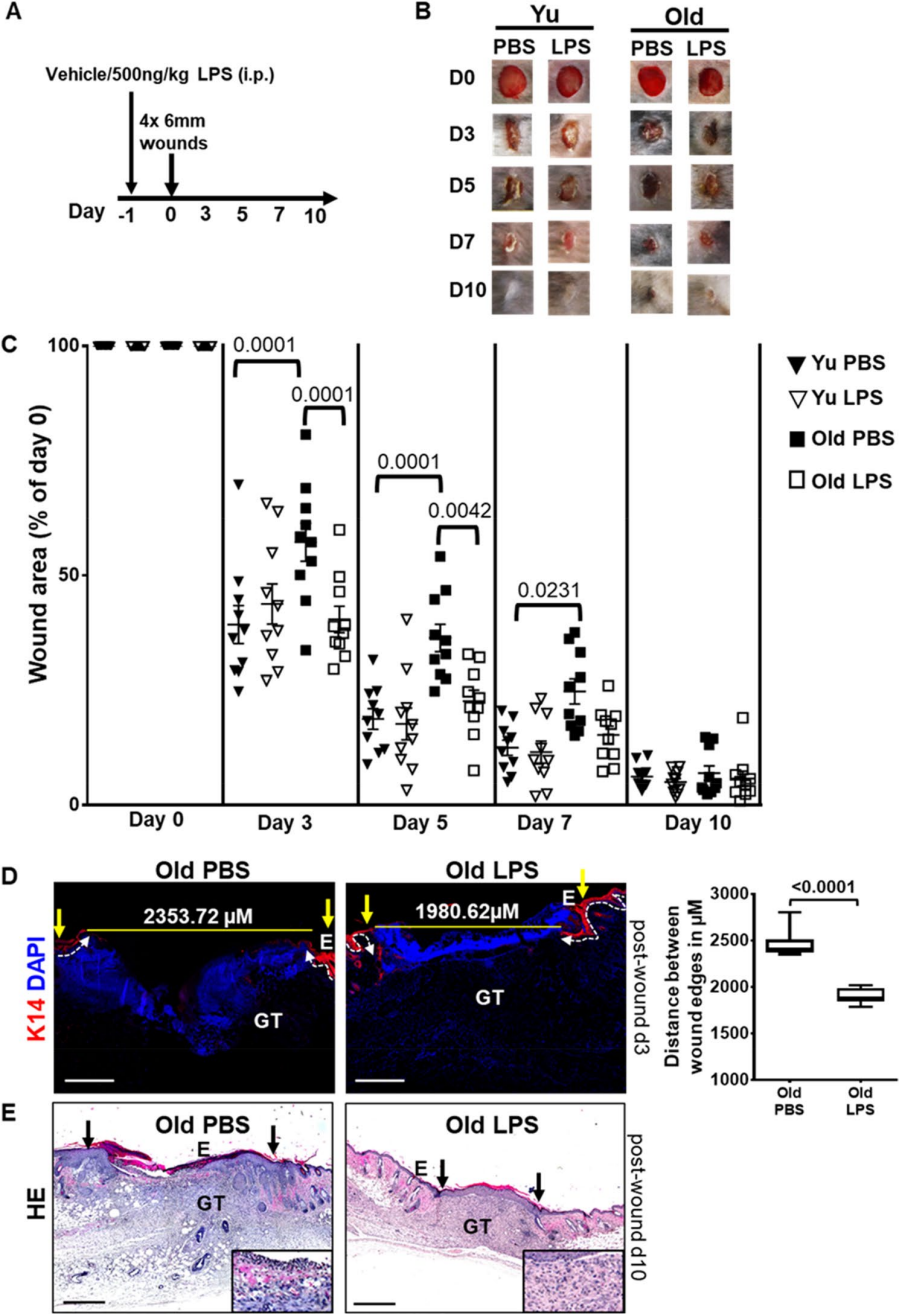
Priming with bacterial LPS accelerates wound repair in old wounds. **A** Scheme showing experimental setting. **B** Representative clinical wound pictures showing time kinetic of wound closure in PBS (vehicle) or LPS challenged young and old mice. **C** Quantification of wound closure over time in PBS (vehicle) or LPS injected young and old mice. One way ANOVA, values are represented as mean ± SEM, *n* = 5. **D** Immunostaining and quantification of K14 (red) depicting the epithelial tongue extending in d3 wounds of PBS (vehicle) or LPS injected old mice wounds. Nuclei stained with DAPI (blue). Stippled line indicates the epidermal dermal junction. Wound edges marked with vertical arrows. E, epidermis; GT, granulation tissue (*n* = 5). Scale bars, 500 μm. Statistical analysis was performed using unpaired t-test, values are represented as mean ± SEM, *n* = 3. **E** Representative H&E photomicrographs depicting wound architecture in PBS (vehicle) or LPS challenged old mice d10 wounds. Wound edges marked with vertical arrows. Scale bars, 500 μm. E, epidermis; GT, granulation tissue (*n* = 5)

Innate immune cells are endowed with the capacity to directly respond to the bacterial cell wall component lipopolysaccharide (LPS) after binding to the toll-like receptor 4 (TLR4) [30, 31]. Currently, it is unknown whether aging may affect LPS priming and subsequent immune response. We tested the hypothesis whether we can achieve rapid sealing of old skin wounds by mobilizing innate immune cells in sufficient number through a low dose of LPS during the early phases of wound healing. For this purpose, we exposed young and old mice to single low dose of LPS (500ng/kg, intraperitoneal injection) or PBS (vehicle), 24 h before inflicting the wounds on back skin (Fig. 2A).

We took advantage of the fact that upon LPS injection in the peritoneal cavity both neutrophils and macrophages are primed [31]. We observed that wounds from young mice heal significantly faster at d3, d5 and d7 as opposed to delayed healing in old mice (Fig. 2B-C). This data are consistent with earlier findings [5, 12]. Interestingly, brief immune stimulation with LPS before wounding, markedly improved healing with significantly reduced wound area in wounds of old mice as compared to wounds of PBS-injected old mice (Fig. 2B-C). Enhanced wound closure of LPS injected old mice was observed at early stages (d3 and d5) of wound healing. These early phases are associated with enhanced infiltration of innate immune cells at the wound site [10]. The faster wound healing in older mice has a key advantage: pathogens can invade the site of injury less efficiently and for a shorter period. Furthermore, this likely prevents prolonged and enhanced chemokine signaling that keeps attracting immune cells.

In case of young mice, which healed at faster rate, LPS challenge did not exert an additional benefit compared to wound closure of young PBS injected mice (Fig. 2B-C). Apparently, wound healing of young mice is already optimized, and a preceding immune stimulus cannot further accelerate wound healing. However, the mechanisms such as TLR responsiveness and immune activation driving age-specific effects of LPS on skin repair remained unresolved. Further improvement of already fast wound healing in young mice - apart from LPS - may require additional currently unknown factors. Age-dependent disease tolerance can also lead to contrasting patterns of tissue response to LPS in young and old wounds [6–8]. In subsequent experiments, we focused investigating the benefits of LPS priming on wound healing of aged mice.

To explore differences between PBS and LPS injected wounds of old mice in more depth, re-epithelialization in d3 wound sections were monitored. Immunostaining of keratin K14 shows marked re-epithelialization with the formation of epithelial tongues towards the wound center in LPS challenged old mice as opposed to less re-epithelialization of wounds in PBS injected old mice (Fig. 2D). These data suggesting an improved migration and/ or proliferation of wound keratinocytes.

Hematoxylin and eosin (HE) stained sections of d10 wounds show that wounds are almost covered by epithelial tongues from opposing sides with complete closure of the wound in LPS treated old mice (Fig. 2E). We also observed significantly reduced epidermal hyperplasia and inflammatory cells accumulation in the newly regenerated aged wounds from LPS as opposed to PBS groups (Fig. 2E). These data highlight an increased responsiveness of wound keratinocytes upon LPS priming.

In aggregate, these findings suggest beneficial effects of LPS pre-conditioning in accelerating tissue repair in wounds of old mice.

### Skin wounds from aged mice swiftly form a physical barrier upon LPS exposure

Surface epithelia provide a critical barrier to the outer environment [23]. Upon tissue damage, outer barrier formed quickly and robustly to protect internal organs from external insults. Resident epithelial cells and immune cells through coordinated efforts control infections and readily respond to barrier breaches [5, 12, 14]. Though aging may impact on underlying immune cell- and keratinocyte functions and, in consequence, may impair barrier formation, this so far has not been dissected in sufficient detail.

**Fig. 3:**
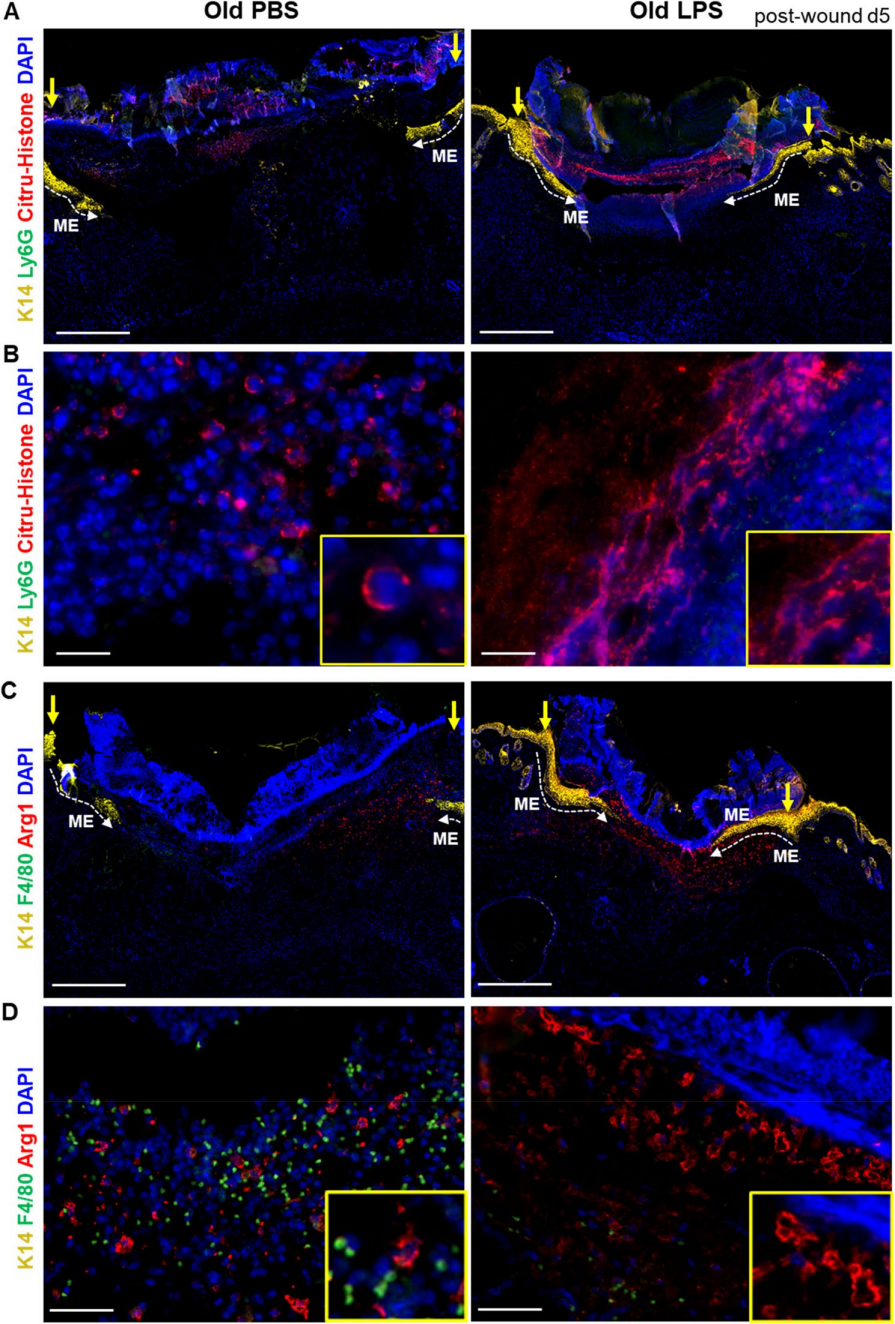
LPS promotes an early cloaking response of neutrophils and Arginase-1 positive macrophage in old wounds. **A**-**B** Immunostaining of citrullinated histone H3 (red), a marker of extracellular trap formation (NET), and Ly6G positive neutrophils (green) in d5 skin wounds of PBS or LPS injected old mice. Wound epidermis is stained with K14 (yellow). Nuclei stained with DAPI (blue). Image A shows an overview of the entire wound, while Image B shows a magnified image. Scale bar: 500 μm in A and 20 μm in B. Stippled white lines indicate the migrating epithelium during re-epithelization. Wound edges marked with yellow vertical arrows. ME: migrating epithelium. **C**-**D** Immunostaining of macrophage markers arginase-1 (red) and F4/80 (green), wound epidermis is stained with K14 (yellow) in d5 wounds of PBS or LPS injected old mice. Nuclei stained with DAPI (blue). Image C shows an overview of the entire wound, while Image D shows a magnified image. Scale bar: 500 μm in C and 50 μm in D. Stippled white lines indicate the migrating epithelium during re-epithelization. Wound edges marked with yellow vertical arrows. Red blood cells exhibiting autofluorescence in green channel. ME: migrating epithelium

Neutrophils are the first immune cells that sense damage and are recruited from the blood circulation to the wound site [32]. In injured tissue they release reactive oxygen species, neutrophil extracellular traps (NETs), neutrophil elastase and myeloperoxidase, proteolytic enzymes collectively fighting pathogens [32]. NETs are typically made of neutrophil chromatin decorated with antimicrobial peptides that trap and potentially kills invading bacteria [33]. To understand the impact of aging on NETs formation, double immunostaining of NETs associated citrullinated histone H3 in combination with Ly6G positive neutrophils was performed. We observed a fine layer of densely packed NETs below the lesion in d5 wounds of young mice forming an organized fibrous barrier with trapped dead cells and tissue debris as opposed to reduced NETs formation in d5 wounds in old mice (Supplementary Fig. 2A-B). Only small fragments of NETs were randomly placed all over granulation tissue in old wounds. Our observation is that LPS challenge in old mice, partially reversed the scant NETs to organized condensed NETs at the wound surface with restoration of the initial physical barrier of wounds as in young mice (Fig. 3A and B and Supplementary Fig. 2C).

We observed two key populations of macrophages expressing F4/80 and arginase-1 on their surface (Fig. 3C-D). Arginase-1 positive macrophages are early responders to injury and recruited from adjacent vessels to the wound site both in wounds of young and old mice (Supplementary Fig. 1B-C). Our observations are consistent with recent findings suggesting hybrid nature of these macrophages expressing both classically activated proinflammatory M1 and alternatively activated tissue resolving M2 markers [5, 22]. Notably, both in young and LPS challenged old wounds, these activated macrophages cover open wounds through their extended membrane protrusions (Fig. 3C-D and Supplementary Fig. 1B-C). Recently, membrane extensions from macrophages were described in resident tissue macrophages in microlesions [14]. Membrane extensions from macrophages physically surround and hide open lesion, and thus, consequently prevent inflammatory cascade [14]. By contrast, wounds from PBS injected old mice are infiltrated mainly with Arg-1 macrophages that are distantly placed at the wound site and partly covering the open lesions (Fig. 3C-D). A continuous layer of DAPI-stained histone DNA fibers was formed on top of the macrophage layer in old LPS-treated wounds, contributing to the physical barrier, these fibrous structures appeared even before the merging of epithelial tongue (Fig. 3C-D). These findings suggesting structural contribution of LPS-primed innate immune cells in physical skin barrier formation and rapid sealing of old wounds. However, the mechanism behind the improvement in wound healing, which arises specifically from systemic inflammatory LPS priming and the localized barrier sealing, remains unresolved. It is also unresolved whether similar to systemic LPS priming, cutaneous LPS injection would benefit wound healing and barrier sealing.

**Fig. 4:**
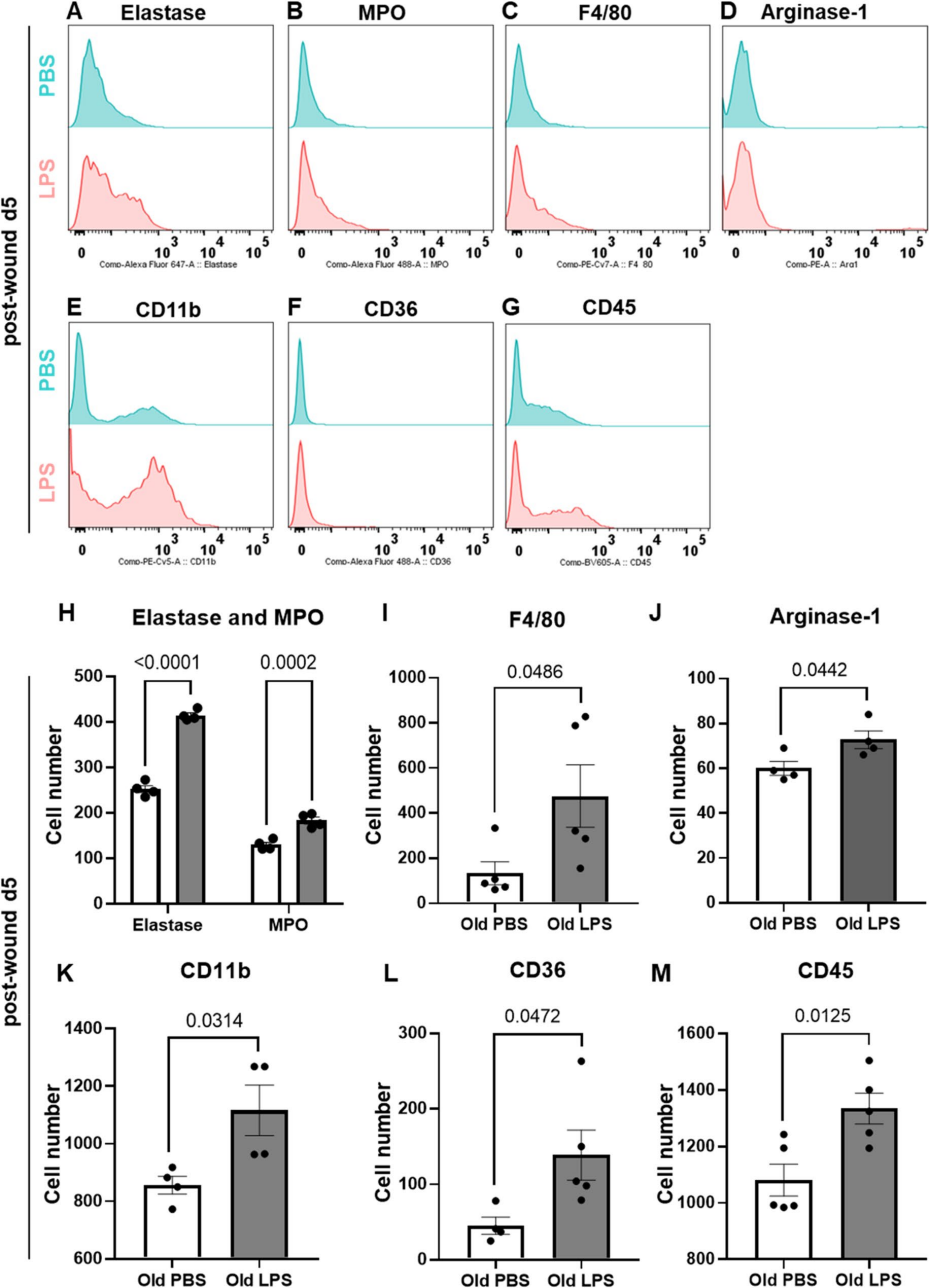
LPS drives infiltration of selected innate immune cells into old wound. **A-G** Flow cytometry analysis of the indicated immune cell populations from d5 wounds that were harvested by enzymatic digestion. Histograms depicting shift in immune cells of old mice wounds following PBS or LPS exposure. **H**-**M** Quantification of indicated populations presented in Fig. 4A. All p-values were obtained from unpaired t-test, values are represented as mean ± SEM, *n* = 3

Employing complementary FACS analysis, we further analyzed immune cells in a cell suspension prepared by enzymatic digestion of d5 wounds from PBS and LPS-treated old mice. Consistent with our findings, we observed increased expression of neutrophil elastase and MPO in old wounds following LPS challenge as opposed to PBS treated old wounds (Fig. 4A-B and H). In addition, our FACS analysis shows that a key macrophage population, including (F4/80, Arg-1 and Cd11b) were significantly increased in d5 wound samples from LPS-primed wounds as compared to PBS (Fig. 4C-E and I-K).

The expression of CD36, a scavenger receptor commonly expressed by phagocytic cells that clears apoptotic cells, remove bacterial and fungal pathogens, was higher in LPS wounds as compared to PBS wounds (Fig. 4F and L). In line with these findings, we observed higher numbers of CD45 expressing hematopoietic cells in LPS wounds as opposed to PBS wounds (Fig. 4G and M). The number of immune cells expressing CD3, CD86 and CD206 remained unchanged following LPS administration (Supplementary Fig. 2D-F). These data suggest that innate immune cells — likely by rapid recruitment or differentiation — occur at higher numbers at the old wound site after low dose of LPS.

LPS injection through intraperitoneal (IP) route in mice triggers a systemic inflammatory response that causes a delayed and less severe inflammatory cascade in the lungs compared to direct lung exposure [34]. We next explored whether cytokine storm induced by single injection of low dose LPS leads to the infiltration of inflammatory cells into the uninjured lungs and old skin.

Histological analysis of old lungs revealed that LPS did not induce any major injuries to alveolar units and changes in lung parenchyma as compared to PBS group (Supplementary Fig. 3A). However, we observed an increased number of activated neutrophils expressing neutrophil elastase upon LPS administration (Supplementary Fig. 3B), while no significant difference was observed in macrophage number in comparison to PBS group (Supplementary Fig. 3C). In addition, we observed no differences in proliferation rate of lung epithelium between two groups suggesting the low dose LPS that we used in our studies did not alter the cellular dynamics (Supplementary Fig. 3D). Quantification of skin immune cells including macrophages (F4/80 and CD206) and T cells (CD3) suggest that LPS did not induce immune cells activity in uninjured skin (Supplementary Fig. 4A-C). Neutrophils numbers were very low in both vehicle and LPS challenged unwounded skin, therefore excluded from the analysis. We did observed significant differences in the expression of pro-inflammatory cytokines such as IL-1β but not in IL-6 between vehicle and LPS treated uninjured skin (Supplementary Fig. 4D-E). These data on uninjured lungs and skin may suggest that the wounding mediated second hit on immune compartment diverted the circulating immunes cells traffic mainly to the injured skin.

Collectively, we interpreted our correlative data that LPS priming may enforce the formation of an early physical wound barrier by combined NET deposition and layered Arg-1 macrophages, physically sequestrating or cloaking pro-inflammatory debris. This potentially prevent dysregulated neutrophil recruitment and the swarming cascade of perpetuated inflammatory cell recruitment in old wounds.

### Suppression of prolonged inflammation in wounds of old mice after LPS priming

**Fig. 5:**
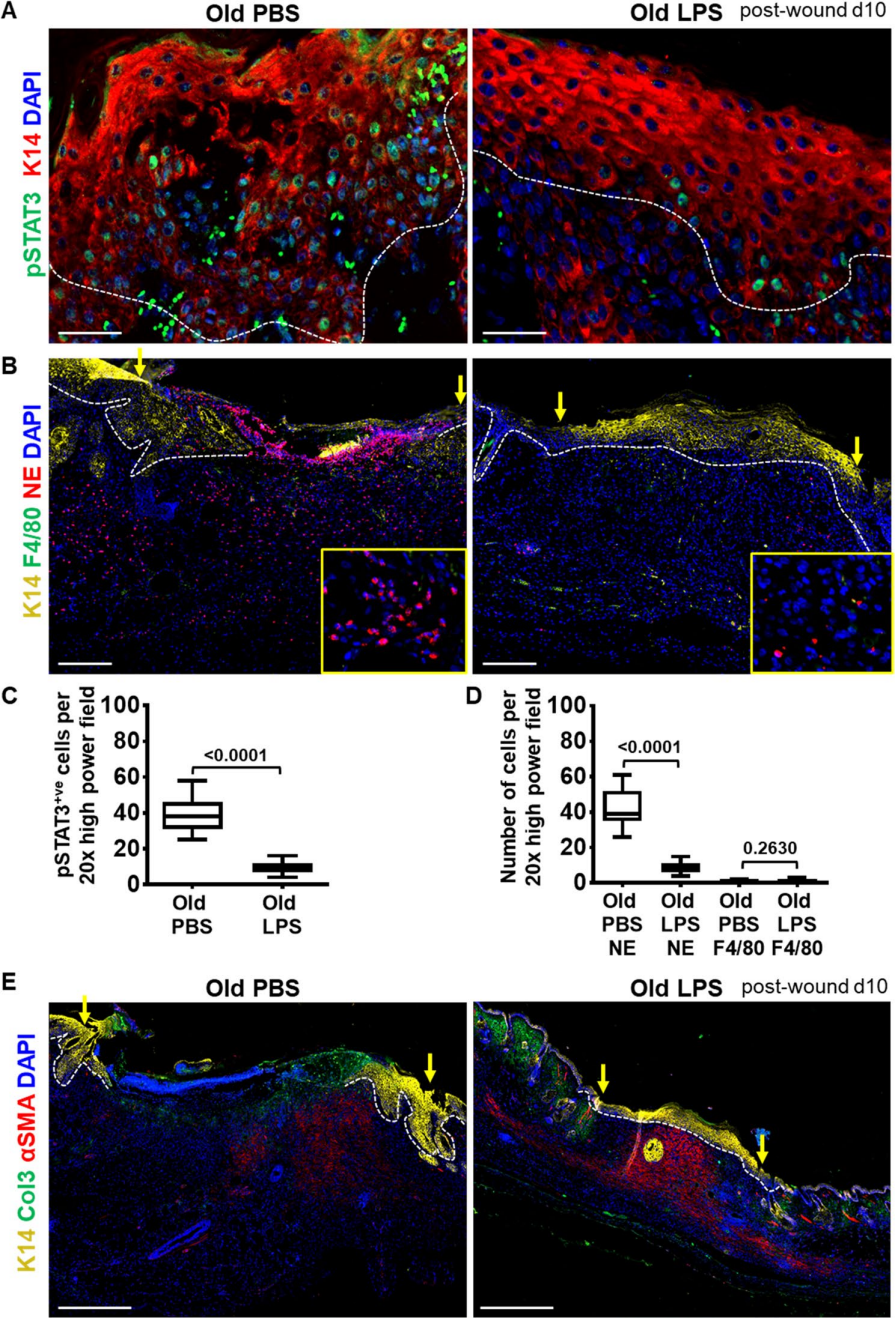
Reduced inflammation in old wounds preceding LPS injection. **A** Immunostaining of pSTAT3 (green), a marker for the pro-inflammatory transcription factor, and of K14 (red), an epidermal marker for basal keratinocytes in 10-day wounds of PBS or LPS injected old mice. Nuclei stained with DAPI (blue). Stippled line indication the epidermal dermal junction. Scale bar: 50 μm. **B** Immunostaining of neutrophil elastase (NE) marking neutrophils (red) and F4/80 (green), a marker for macrophages in wounds of PBS or LPS injected old mice. Wound epidermis is stained with K14 (yellow) indicating wound closure. Nuclei stained with DAPI (blue). Scale bar: 200 μm. **C** Quantification of pSTAT3 positive keratinocytes shown in Fig. 5A. Statistical analysis was performed using unpaired t-test, values are represented as mean ± SEM, *n* = 3. **D** Quantification of neutrophils (NE) and macrophages (F4/80) shown in Fig. 5B. Statistical analysis was performed using unpaired t-test, values are represented as mean ± SEM, *n* = 3. **E** Immunostaining of αSMA (red), a marker for dermal myofibroblasts that regulates dermal contractility and ECM remodeling, and Collagen 3 (green), a major component of the ECM, in wounds of PBS or LPS injected old mice. K14 (yellow), an epidermal marker for basal keratinocytes. Scale bar: 500 μm. Nuclei stained with DAPI (blue)

To elucidate whether immune pre-conditioning by LPS can be exploited to combat persistent inflammation in wounds of aged mice, we next investigated the inflammatory response. We are interested whether the impaired re-epithelialization in wounds of old mice is driven by persistent Inflammation and whether this can be reversed by LPS priming. Indeed, LPS treatment markedly abrogated the expression of pSTAT3 in d10 wound keratinocytes when compared to PBS treated old mice (Fig. 5A and C). This is of interest as pSTAT3 is usually upregulated in inflamed tissues [17, 35]. Although more markers are needed to establish inflammation as pSTAT3 is also upregulated in keratinocytes during re-epithelialization [12]. Of note, the LPS dependent restoration of skin barrier markedly suppress the unrestrained neutrophil recruitment in late wounds of aged mice, which quickly respond upon barrier breaches and swarm into injured tissue (Fig. 5B and D).

Priming with LPS, likely by rapid barrier restoration effectively attenuates chemotaxis and suppresses continuous inflow and persistence of neutrophils at the wound site. This not only breaks the vicious cycle of inflammation, but also prevents the enhanced release of neutrophil proteolytic enzymes contributing to enhanced tissue damage at the wound site in aged mice. We here highlight the delayed but persistent neutrophil activation as the major cause of wound healing delay in aged mice. By contrast, the number and expression of inflammation resolving F4/80 macrophages that significantly increased in d10 young wounds as opposed to old wounds (Supplementary Fig. 1B-C), did not markedly change in number in wounds of aged mice even upon LPS stimulation (Fig. 5B and D). Suggesting that the rescue of F4/80 macrophages function and activity in old wounds most likely need an additional intervention or requires different treatment schedule of LPS.

Consistent with our earlier finding which suggest enhanced re-epithelialization in LPS treated old wounds, the expression of K10 was markedly enhanced at wound periphery as well as in the center (Supplementary Fig. 5A), indicating enhanced epidermal differentiation towards a functional epidermal barrier.

Finally to investigate whether LPS has any influence on dermal extracellular matrix deposition in old chronic wounds. Therefore, we analyzed the expression of matrix producing αSMA^+^ myofibroblasts and collagen in 10 days old wound from LPS and control group. The αSMA^+^ myofibroblasts are unique type of injury activated cutaneous fibroblasts that promote healing in chronic wounds by facilitating contractility, reepithelization, and vascularization [36]. We observed strong accumulation of αSMA^+^ myofibroblasts in the entire region of the regenerating scar tissue from LPS, while a partial expression was observed in the vehicle group (Fig. 5E and Supplementary Fig. 5B). Type III collagen (Col3), highly abundant type of collagen in the body, plays a crucial role in tissue maintenance and repair [37]. However, we did not observe differences in collagen deposition between vehicle and LPS wounds (Fig. 5E).

**Fig. 6:**
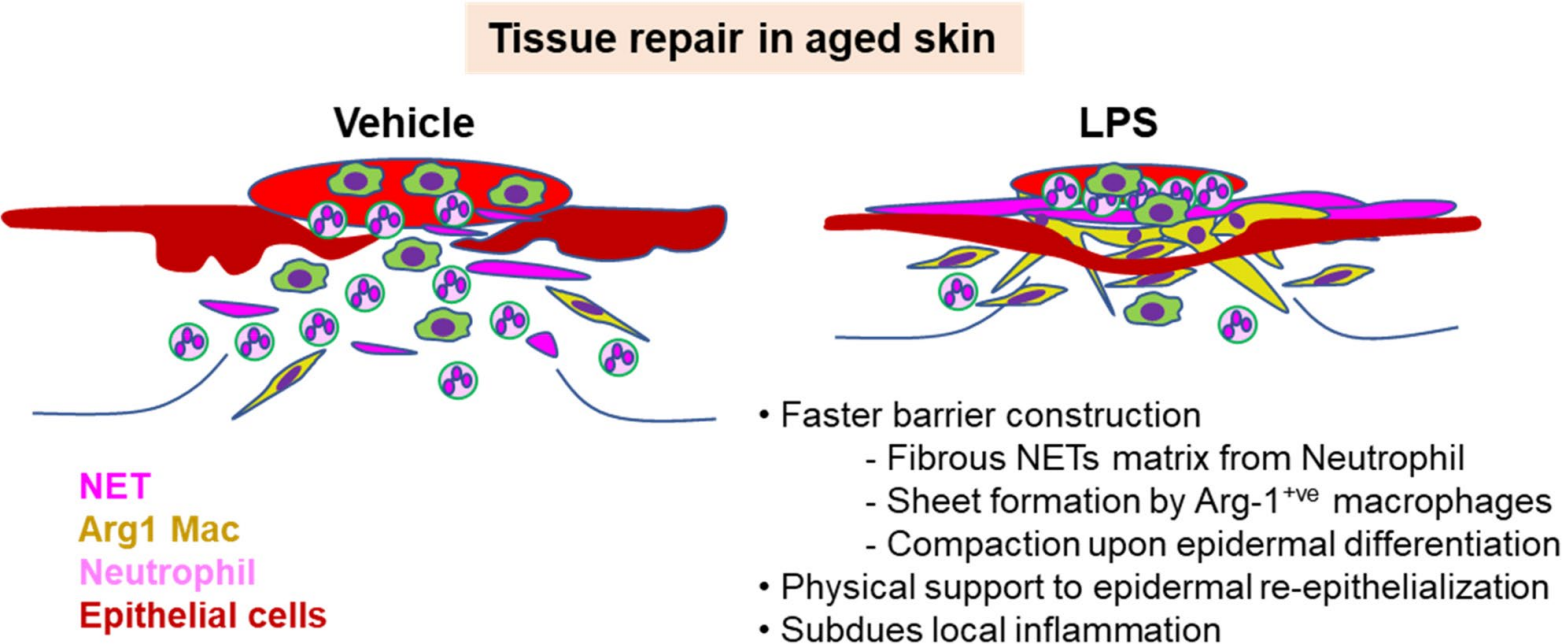
Graphical summary

Priming with LPS before skin injury enhances wound repair in old mice. A short pulse of LPS before skin injury triggers faster closure of open wounds through induction of extracellular traps from neutrophils which fight infection, subsequent control of neutrophil activation by Arg-1 positive macrophage-mediated cloaking response, and the formation of a compact epidermal barrier consisting of differentiated wound keratinocytes. The combination of these interrelated events resolves inflammation by inhibiting continuous recruitment and activation of neutrophils which- if dysregulated as in aged skin wounds - perpetuates and aggravates local tissue damage.

In aggregate, our findings highlight that LPS mediated activation of innate immune system not only reinforce physical barrier built by the structural components that prevent invasion but also adds a biochemical barrier that provides antimicrobial defense (Fig. 6). This subsequently support keratinocyte activity and suppresses propagation of inflammation and eventually promote wound repair in aged skin.

## Discussion

In this study we have systematically delineated the unique advantage of immune priming in suppressing inflammation and enhancing tissue repair in the skin of aged mice. Our study provides a framework for understanding fundamental cellular events that govern wound healing process and in the long term holds promise for refined therapy of wounds in elderly.

Injured tissue reacts swiftly and exert a strong inflammatory response to protect the organism from invading pathogen [8, 10]. This process is highly regulated and inflammation resolved in timely manner following injury. However, in case inflammation persists over a longer period, this could be detrimental to the surrounding tissue as reported during aging or in other chronic wound healing disorders preferentially occurring in old adults [4, 5, 12, 13, 19, 38]. Although great progress has been made to understand the mechanisms regulating inflammation [39, 40], the knowledge of checkpoints counteracting inflammation during the late phases of tissue repair is still not fully understood. Several lines of evidence indicate that checkpoints critical for resolution of inflammation are compromised in aging and may lead to inflammatory wound state [1, 3, 5]. This study was therefore designed to explore the benefits of immune priming on age- related chronic wounds and to investigate whether transient immune stimulation can re-activate checkpoint pathway that efficiently curb inflammation. Moreover, it remains to be elucidated how phasic repair response is decoupled during aging.

The key finding of our study is that the bacterial wall component LPS - known to enforce pro-inflammatory reactions - enhances skin repair in aged mice. LPS binds to the TLR4 receptor on immune cells, and then activates the downstream AP-1, NF-κB Akt, and MAPK pathways [41]. Beneficial effects of LPS on acceleration of wound healing in old mice are attributed to different cascade of events. First, the formation of an early barrier at the site of injury through deposition of NETs in an organized manner. This suggesting that NET not only trap bacteria as conventionally suggested but also by passively contributing to rapid barrier formation that prevents further invasion into sterile tissue. Secondly, Arg-1 hybrid macrophages which are recruited concomitantly add another layer of defense by physically surrounding the open lesions with their membrane protrusion, and thereby interrupt the vicious cycle of persisting neutrophil influx. These findings highlight the link between innate immune cells and structural constituents including DNA histone NETs and membrane protrusions. This association has previously been reported that neutrophils formed a ring of collagen matrix around the wound following injury and thereby physically shielded the injured host from the outside world [42]. Macrophages also reported to participate in barrier organization by forming fibrin clot that physically trap pathogen and seal wound within serosal cavities [43] or by modulating collagen composition via activating mesenchyme [44]. Indeed, we observed strong induction of αSMA produced by myofibroblasts in the old wounds primed with LPS. In wounds, myofibroblasts not only produce ECM but also provides mechanical strength to the tissue [36].

Thirdly, separation of apoptotic neutrophils and tissue debris by these protective layers prevent unrestrained neutrophils recruitment and activation at the injured site and hence effectively halt inflammation propagation and tissue damage. Finally, reduced local inflammation not only expedites the re-epithelialization process, but also promotes terminal keratinocyte differentiation of re-epithelializing keratinocytes, which eventually tightly seals the wound by epidermal barrier formation.

Our findings suggest a contribution of macrophages in physical barrier formation are in line with earlier report [14]. These authors earlier report on a similar function of resident tissue macrophages in protecting laser-induced single cell death microlesions, though the role of peripheral and tissue resident macrophages in large injuries was not investigated. According to their findings resident tissue macrophages involved receptor RAGE and respond to a variety of danger associated molecular patterns DAMPs, such as HMGB1 and S100, which generate the mature cloaking response [14, 32]. Our findings on the role of these macrophages in full thickness wounds is unprecedented and help to advance our understanding on Arg-1 positive macrophages especially in the context of aging. As such, Arg-1 positive macrophages are randomly distributed in a distant neighborhood of wounds of aged mice. However, LPS priming helps them to organize in a continuous layer attributing to barrier compaction at the surface of wounds, and thus they likely contribute to the neutrophil suppressing response.

In the present study, we tested the concept that immune cell priming can benefit aged skin wounds. One of limitations of our study is that the causal role of NETs and macrophage-derived structures in barrier formation has not been fully elucidated in terms of the underlying mechanisms. Future studies are needed to determine the precise contribution of systemic versus local LPS mediated preconditioning of immune cell on barrier formation and tissue remodeling. Topical application of LPS to wounds may be a better approach as compared to systemic LPS administration as it likely would reduces adverse side effects. As such LPS can lead to lung complications, gut lesions, neurodegenerative diseases, cytokine storm and septic shock in the patients [45, 46]. If the LPS dose is not carefully calibrated, it can be life-threatening to patients as humans are much more sensitive than mice. Unrestrained inflammatory priming in aged tissue may risks accelerating “inflammaging,” a state of chronic, low-grade inflammation that drives tissue damage, functional decline, and age-related diseases [47]. In perspective, for translation of our clinically relevant observation for non-healing wounds of old human adults, TLR-4 agonists with less side effects would be a safer alternative to LPS to attenuate the aging immune system.

Consistent with earlier reports [5, 12, 13], we also observed a different cellular dynamic in aged tissue undergoing repair. Delayed healing observed in elderly, most likely is due to both intrinsic and extrinsic factors which negatively impact the behavior of innate immune cells. Also disrupt the coordination between different cellular component, production of ECM proteins, restoration of tissue architecture and formation of anatomical barriers. Age associated cellular senescence further deteriorate the healing response [3, 13]. Immune stimulation sensitizes cells of different origin and improves their function through enhancing their chromatin accessibility, where transcription factor binds and initiate gene transcription [17, 18, 30, 31].

## Conclusions

Our results extend the benefits of immune priming in treating age-associated chronic wounds through restoring the microanatomy of damaged tissue that counteracts unrestrained bouts of inflammations. We here presented a concept that through transient immune challenge aged tissue can effectively be prepared to respond in case of skin injury more favorably. Collectively, these findings provide intriguing insights into potential therapeutic interventions with immune disorders and compromised barriers, including for obesity, diabetes and aging-related wound healing disorders.

## Supplementary Information

Supplementary Material 1. Supplementary Figure 1. Aging impairs sealing of open wounds. (A) Quantification of neutrophils (NE) and macrophages (F4/80) shown in Figure 1D. Statistical analysis was performed using unpaired t-test, values are represented as mean ± SEM, *n*=3 (B) Immunostaining of macrophage markers Arginase-1 (red) and F4/80 (green), wound epidermis is stained with K14 (yellow) in young and old mouse skin wounds. Nuclei stained with DAPI (blue). Scale bar: 200µm. (C) Quantification of F4/80 and Arginase-1 expressing macrophages depicted in Figure S1B. Statistical analysis was performed using unpaired t-test, values are represented as mean ± SEM, *n*=3. Supplementary Figure 2. Neutrophil function declines during aging. (A) Immunostaining of citrullinated histone H3 (red), a marker of extracellular trap formation (NETs), and Ly6G positive neutrophils (green) in skin wounds of young and old mice. Nuclei stained with DAPI (blue). Scale bar: 200µm. (B) Quantification of NETs stained with citrullinated histone H3 (red) in Figure S2A. Statistical analysis was performed using unpaired t-test, values are represented as mean ± SEM, *n*=3.(C) Quantification of NETs stained with citrullinated histone H3 (red) in Figure 3A. (D-F) Quantification of indicated immune cell populations from d5 wounds by flow cytometry. Old mice were treated either with PBS or LPS. Supplementary Figure 3. Intraperitoneally injected LPS mild immune response in old lungs. (A) Representative H&E photomicrographs of lungs from old mice exposed to PBS or LPS. Lungs were collected post 10 days skin wounding. Scale bar, 500µM. (B) Representative immunostaining microphotographs and quantification of NE (red) and neutrophil marker Ly6G (green) in lungs sections collected post 10 days skin wounding of either LPS or vehicle treated old mice. Cell nuclei are stained with DAPI (blue). Scale bar, 50µM. (C) Representative immunostaining microphotographs and quantification of Arg-1 (red) and macrophage marker F4/80 (green) in lungs sections collected post 10 days skin wounding of either LPS or vehicle treated old mice. Cell nuclei are stained with DAPI (blue). Scale bar, 50µM. (D) Representative immunostaining microphotographs and quantification of K14 (red) and proliferation marker Ki-67 (green) in lungs sections collected post 10 days skin wounding of either LPS or vehicle treated old mice. Cell nuclei are stained with DAPI (blue). Scale bar, 50µM. Supplementary Figure 4. LPS does not trigger immune cells infiltration in unwounded skin. (A) Representative immunostaining microphotographs and quantification of pan macrophage marker F4/80, (B) M2 macrophage marker CD206 and (C) T cell marker CD3 in green from unwounded old skin sections collected post 24h of LPS or vehicle challenge. Neutrophil are stained with NE (red) and epidermis is marked with K14 (purple). Nuclei stained with DAPI (blue). Stippled line indication the epidermal dermal junction. Scale bar, 50µM. (D) Representative immunostaining images and quantification of cytokine IL-1β and (E) IL-6 (green) in unwounded old skin sections collected post 24h of LPS or vehicle challenge. Neutrophil are stained with NE (red) and epidermis is marked with K14 or α6 β4 integrin (purple). Nuclei stained with DAPI (blue). Stippled line indication the epidermal dermal junction. Scale bar, 50µM. Supplementary Figure 5. LPS promotes keratinocyte migration and differentiation during wound healing. (A) Immunostaining of K14 (red), a marker for basal keratinocytes, and K10 (green), a marker for differentiated keratinocytes in wounds of PBS or LPS injected old mice. Scale bar: 200µm. Nuclei stained with DAPI (blue). (B) Quantification of αSMA immunostaining (red) shown in Figure 5E.

## Data Availability

No datasets were generated or analysed during the current study.
