# Supplementary material for: Transient immunostimulation with LPS promotes tissue repair in aged skin
Source: Immun Ageing. 2026 May 7;23:17. doi: 10.1186/s12979-026-00570-y (PMC13154484; doi:10.1186/s12979-026-00570-y)
Supplement: Supplementary file 1 — Supplementary Material 1. Supplementary Figure 1. Aging impairs sealing of open wounds. (A) Quantification of neutrophils (NE) and macrophages (F4/80) shown in Figure 1D. Statistical analysis was performed using unpaired t-test, values are represented as mean ± SEM, n=3 (B) Immunostaining of macrophage markers Arginase-1 (red) and F4/80 (green), wound epidermis is stained with K14 (yellow) in young and old mouse skin wounds. Nuclei stained with DAPI (blue). Scale bar: 200µm. (C) Quantification of F4/80 and Arginase-1 expressing macrophages depicted in Figure S1B. Statistical analysis was performed using unpaired t-test, values are represented as mean ± SEM, n=3. Supplementary Figure 2. Neutrophil function declines during aging. (A) Immunostaining of citrullinated histone H3 (red), a marker of extracellular trap formation (NETs), and Ly6G positive neutrophils (green) in skin wounds of young and old mice. Nuclei stained with DAPI (blue). Scale bar: 200µm. (B) Quantification of NETs stained with citrullinated histone H3 (red) in Figure S2A. Statistical analysis was performed using unpaired t-test, values are represented as mean ± SEM, n=3.(C) Quantification of NETs stained with citrullinated histone H3 (red) in Figure 3A. (D-F) Quantification of indicated immune cell populations from d5 wounds by flow cytometry. Old mice were treated either with PBS or LPS. Supplementary Figure 3. Intraperitoneally injected LPS mild immune response in old lungs. (A) Representative H&E photomicrographs of lungs from old mice exposed to PBS or LPS. Lungs were collected post 10 days skin wounding. Scale bar, 500µM. (B) Representative immunostaining microphotographs and quantification of NE (red) and neutrophil marker Ly6G (green) in lungs sections collected post 10 days skin wounding of either LPS or vehicle treated old mice. Cell nuclei are stained with DAPI (blue). Scale bar, 50µM. (C) Representative immunostaining microphotographs and quantification of Arg-1 (red) and macrophage marker [file 12979_2026_570_MOESM1_ESM.docx]

**Supplementary Information**

**
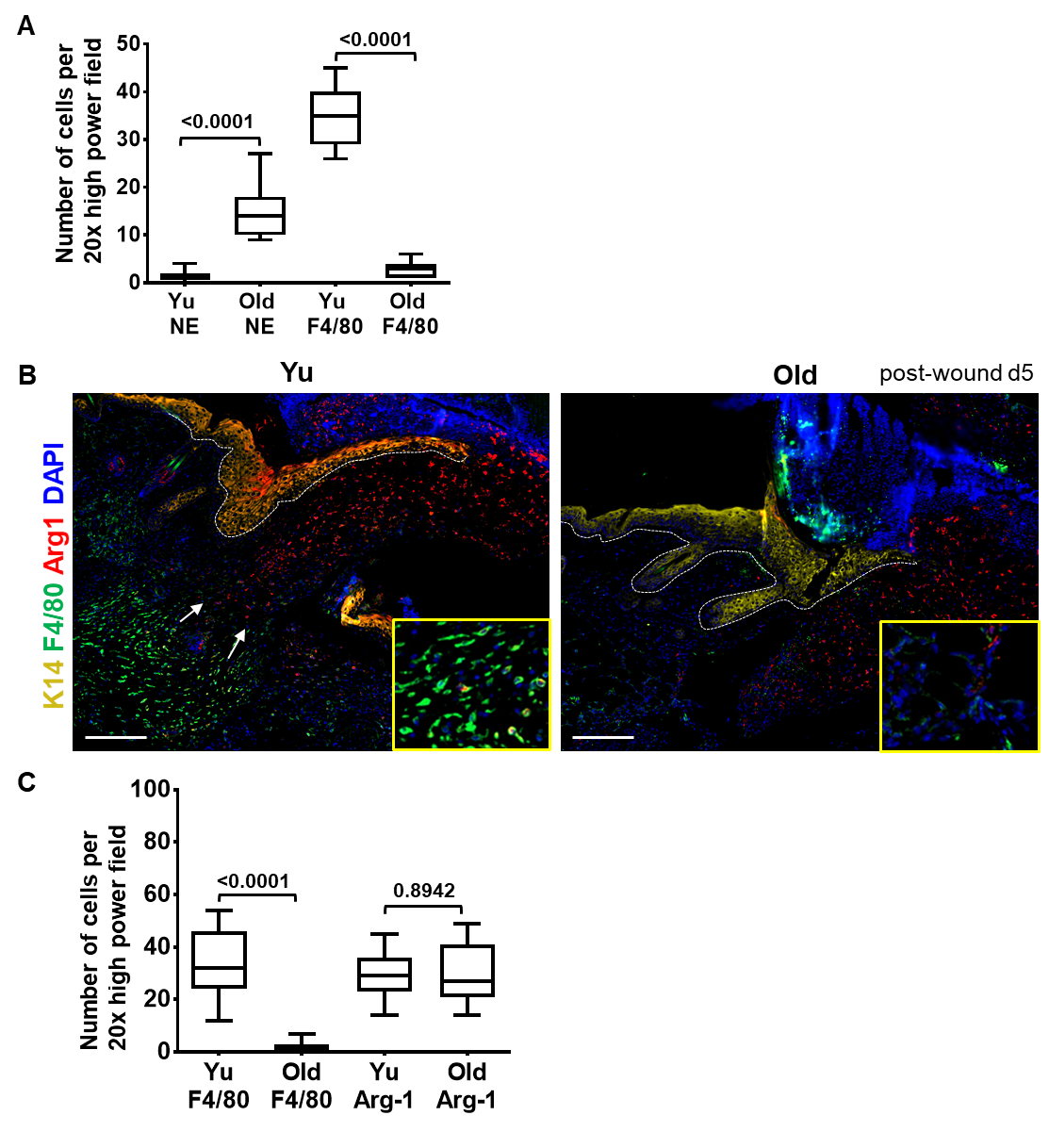
**

**Supplementary Figure 1. Aging impairs sealing of open wounds.**

**(A)** Quantification of neutrophils (NE) and macrophages (F4/80) shown in Figure 1D. Statistical analysis was performed using unpaired t-test, values are represented as mean ± SEM, n=3

**(B)** Immunostaining of macrophage markers Arginase-1 (red) and F4/80 (green), wound epidermis is stained with K14 (yellow) in young and old mouse skin wounds. Nuclei stained with DAPI (blue). Scale bar: 200µm.

**(C)** Quantification of F4/80 and Arginase-1 expressing macrophages depicted in Figure S1B. Statistical analysis was performed using unpaired t-test, values are represented as mean ± SEM, n=3.

**
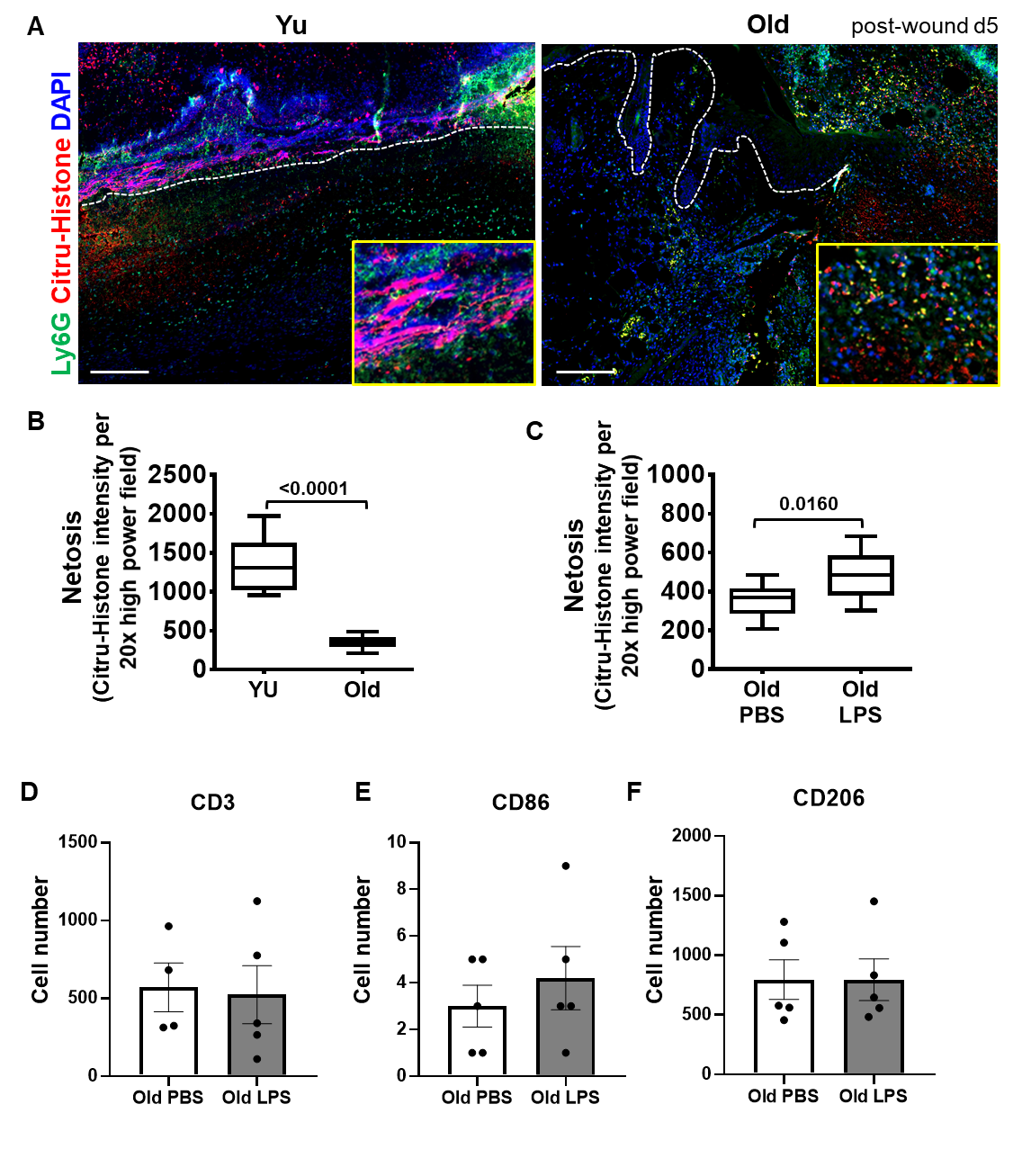
**

**Supplementary Figure 2. Neutrophil function declines during aging.**

**(A)** Immunostaining of citrullinated histone H3 (red), a marker of extracellular trap formation (NETs), and Ly6G positive neutrophils (green) in skin wounds of young and old mice. Nuclei stained with DAPI (blue). Scale bar: 200µm.

**(B)** Quantification of NETs stained with citrullinated histone H3 (red) in Figure S2A. Statistical analysis was performed using unpaired t-test, values are represented as mean ± SEM, n=3.

**(C)** Quantification of NETs stained with citrullinated histone H3 (red) in Figure 3A.

**(D-F)** Quantification of indicated immune cell populations from d5 wounds by flow cytometry. Old mice were treated either with PBS or LPS.


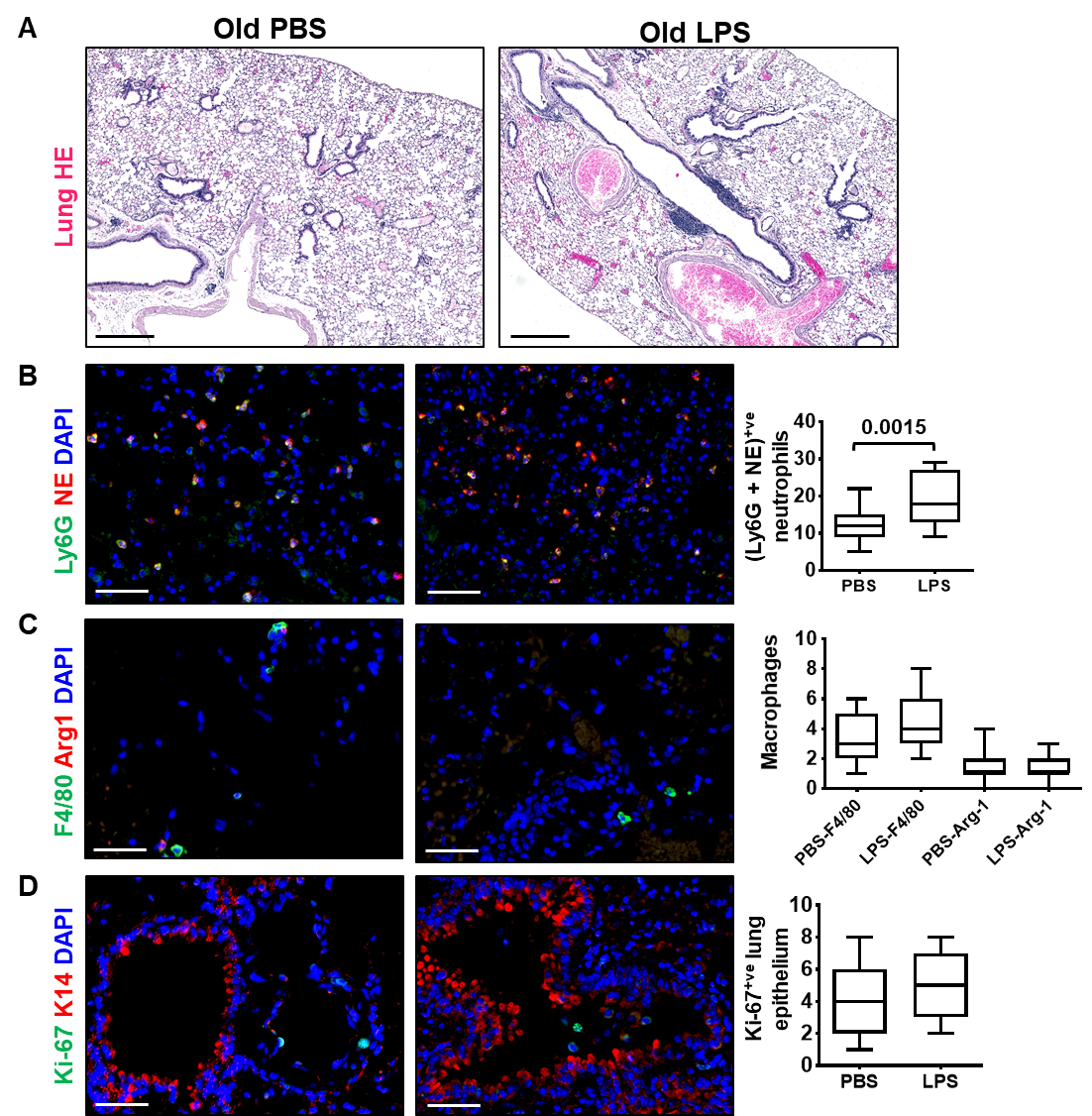


**Supplementary Figure 3. Intraperitoneally injected LPS mild immune response in old lungs.**

**(A)** Representative H&E photomicrographs of lungs from old mice exposed to PBS or LPS. Lungs were collected post 10 days skin wounding. Scale bar, 500µM.

**(B)** Representative immunostaining microphotographs and quantification of NE (red) and neutrophil marker Ly6G (green) in lungs sections collected post 10 days skin wounding of either LPS or vehicle treated old mice. Cell nuclei are stained with DAPI (blue). Scale bar, 50µM.

**(C)** Representative immunostaining microphotographs and quantification of Arg-1 (red) and macrophage marker F4/80 (green) in lungs sections collected post 10 days skin wounding of either LPS or vehicle treated old mice. Cell nuclei are stained with DAPI (blue). Scale bar, 50µM.

**(D)** Representative immunostaining microphotographs and quantification of K14 (red) and proliferation marker Ki-67 (green) in lungs sections collected post 10 days skin wounding of either LPS or vehicle treated old mice. Cell nuclei are stained with DAPI (blue). Scale bar, 50µM.


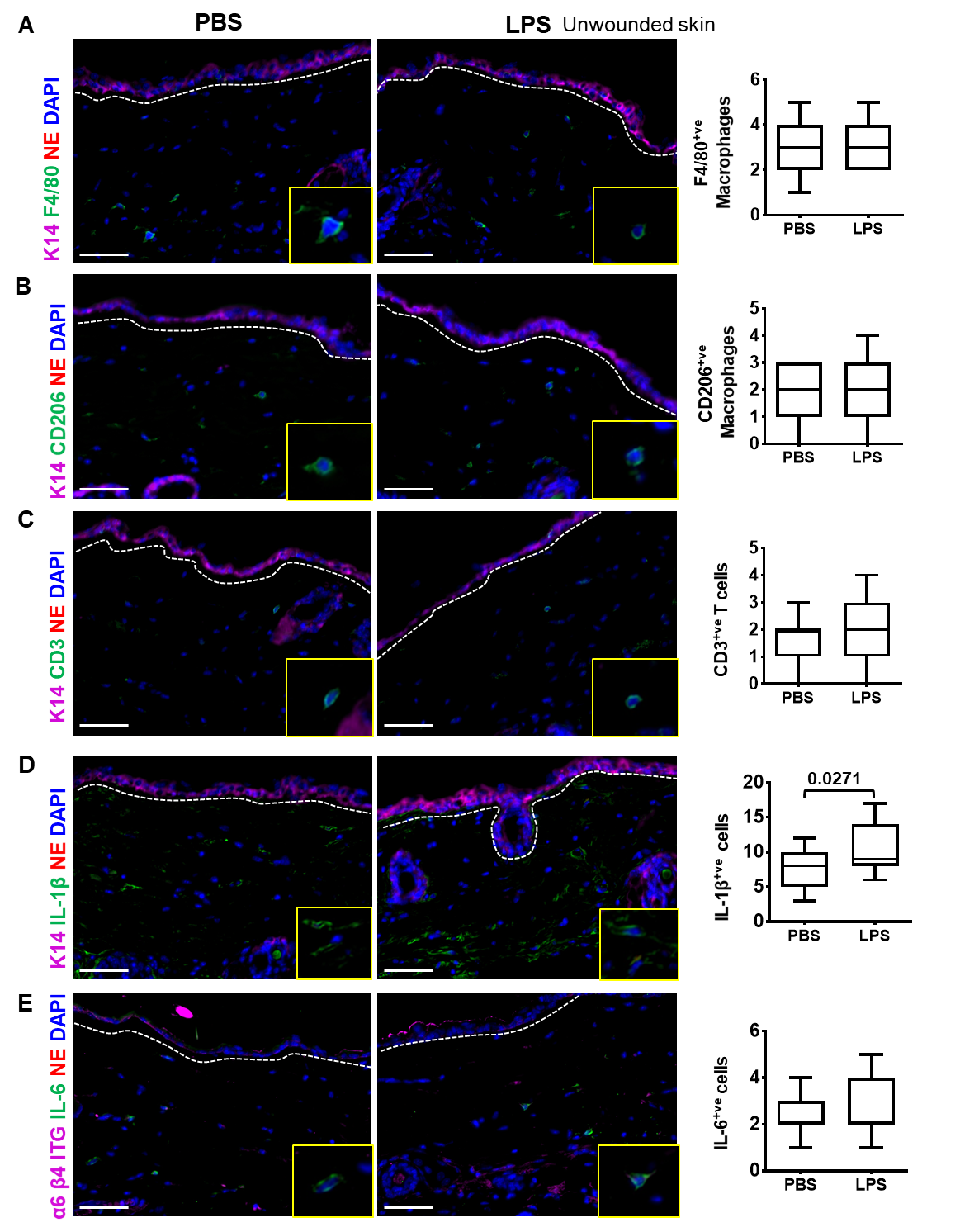


**Supplementary Figure 4. LPS does not trigger immune cells infiltration in unwounded skin.**

**(A)** Representative immunostaining microphotographs and quantification of pan macrophage marker F4/80, **(B)** M2 macrophage marker CD206 and **(C)** T cell marker CD3 in green from unwounded old skin sections collected post 24h of LPS or vehicle challenge. Neutrophil are stained with NE (red) and epidermis is marked with K14 (purple). Nuclei stained with DAPI (blue). Stippled line indication the epidermal dermal junction. Scale bar, 50µM.

**(D)** Representative immunostaining images and quantification of cytokine IL-1β and **(E)** IL-6 (green) in unwounded old skin sections collected post 24h of LPS or vehicle challenge. Neutrophil are stained with NE (red) and epidermis is marked with K14 or α6 β4 integrin (purple). Nuclei stained with DAPI (blue). Stippled line indication the epidermal dermal junction. Scale bar, 50µM.


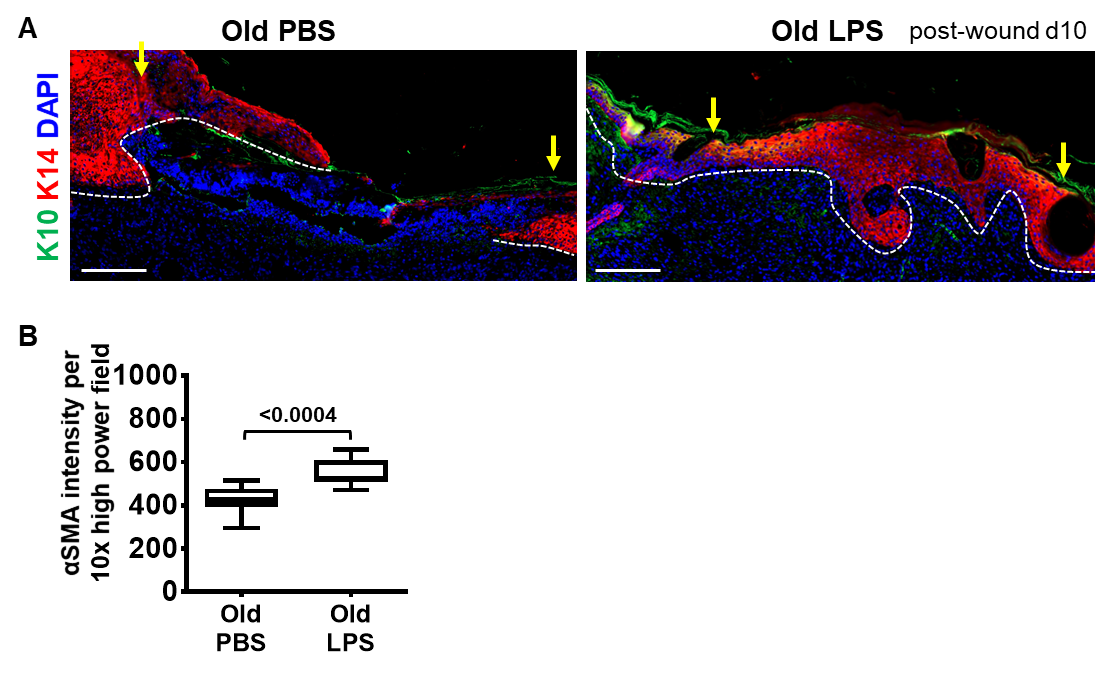


**Supplementary Figure 5. LPS promotes keratinocyte migration and differentiation during wound healing.**

**(A)** Immunostaining of K14 (red), a marker for basal keratinocytes, and K10 (green), a marker for differentiated keratinocytes in wounds of PBS or LPS injected old mice. Scale bar: 200µm. Nuclei stained with DAPI (blue).

**(B)** Quantification of αSMA immunostaining (red) shown in Figure 5E.
